# Functional Independence Assessment in Children and Adolescents with Achondroplasia: A Multicenter Cross-Sectional Study Using the WeeFIM Scale

**DOI:** 10.3390/diagnostics15192532

**Published:** 2025-10-07

**Authors:** Chung-Lin Lee, Hung-Hsiang Fang, Chih-Kuang Chuang, Dau-Ming Niu, Ju-Li Lin, Mei-Chyn Chao, Yen-Yin Chou, Pao Chin Chiu, Chia-Chi Hsu, Tzu-Hung Chu, Yin-Hsiu Chien, Huei-Ching Chiu, Ya-Hui Chang, Yuan-Rong Tu, Yun-Ting Lo, Hsiang-Yu Lin, Shuan-Pei Lin

**Affiliations:** 1 Department of Pediatrics, MacKay Memorial Hospital, Taipei 10449, Taiwan; clampcage@gmail.com (C.-L.L.); spty871029@hotmail.com (H.-H.F.); g880a01@mmh.org.tw (H.-C.C.); wish1001026@gmail.com (Y.-H.C.); 2 Institute of Clinical Medicine, National Yang-Ming Chiao-Tung University, Taipei 112304, Taiwan; 3 International Rare Disease Center, MacKay Memorial Hospital, Taipei 10449, Taiwan; andy11tw.e347@mmh.org.tw; 4 Department of Medicine, Mackay Medical University, New Taipei City 252, Taiwan; 5 Mackay Junior College of Medicine, Nursing and Management, Taipei 112021, Taiwan; 6 Department of Pediatrics, Tri-Service General Hospital, National Defense Medical Center, Taipei 114202, Taiwan; 7 Division of Genetics and Metabolism, Department of Medical Research, MacKay Memorial Hospital, Taipei 10449, Taiwan; mmhcck@gmail.com (C.-K.C.); likemaruko@hotmail.com (Y.-R.T.); 8 College of Medicine, Fu-Jen Catholic University, Taipei 242062, Taiwan; 9 Department of Pediatrics, Taipei Veterans General Hospital, Taipei 11217, Taiwan; dmniu1111@yahoo.com.tw; 10 Department of Pediatrics, Division of Genetics and Pediatric Endocrinology, Linkou Chang Gung Memorial Hospital, Taoyuan 333, Taiwan; jllin001@gmail.com; 11 Department of Pediatrics, Changhua Christian Children’s Hospital, Changhua 500, Taiwan; mcchao1@gmail.com; 12 Department of Pediatrics, National Cheng Kung University Hospital, Tainan 704, Taiwan; yenyin@mail.ncku.edu.tw; 13 Department of Pediatrics, Kaohsiung Veterans General Hospital, Kaohsiung 813414, Taiwan; paochinped333@gmail.com; 14 Division of Pediatric Genetics and Metabolism, Children’s Medical Center, Taichung Veterans General Hospital, Taichung 407219, Taiwan; chiacky@vghtc.gov.tw; 15 Department of Pediatrics, China Medical University Hsinchu Hospital, Hsinchu 302, Taiwan; mno4.chu@gmail.com; 16 Department of Medical Genetics, National Taiwan University Hospital, Taipei 100225, Taiwan; chienyh@ntu.edu.tw; 17 Department of Medical Research, China Medical University Hospital, China Medical University, Taichung 40447, Taiwan; 18 Department of Infant and Child Care, National Taipei University of Nursing and Health Sciences, Taipei 108306, Taiwan

**Keywords:** achondroplasia, functional independence, WeeFIM, pediatric rehabilitation, daily living activities

## Abstract

**Background/Objectives:** Achondroplasia is the most common skeletal dysplasia, affecting 1 in 25,000 births. Limited research exists on the assessment of functional independence using standardized tools in children and adolescents with achondroplasia. The WeeFIM scale provides a comprehensive evaluation of daily living skills across multiple functional domains. This study aimed to assess the functional independence levels in children and adolescents with achondroplasia using WeeFIM and analyze functional capabilities. **Methods:** This multicenter cross-sectional study included 46 participants aged 6–18 years with confirmed achondroplasia. Data were collected through standardized WeeFIM assessments from medical centers and online surveys (2021–2024). WeeFIM evaluates 18 functional items across 3 domains: self-care (8 items), mobility (5 items), and cognition (5 items), scored 1–7 (complete dependence to independence). **Results:** Participants included 26 males (56.5%) and 20 females (43.5%). Most (78.3%) were diagnosed during infancy. The mean functional scores were highest for cognition (34.0/35, 97.1%), followed by self-care (51.2/56, 91.4%) and mobility (31.5/35, 90.0%). Most participants achieved near-complete independence in cognitive functions. Mobility tasks, particularly stair climbing and bathtub transfers, showed the greatest challenges. Functional independence increased with age, with significant improvements during early childhood to adolescence transition. **Conclusions:** Children and adolescents with achondroplasia demonstrate high functional independence across daily activities, with cognitive abilities largely unaffected. Although specific mobility challenges exist, most participants achieve independence with appropriate accommodations. These findings provide valuable baseline data for clinical care planning and support optimistic functional outcomes for pediatric patients with achondroplasia.

## 1. Introduction

Achondroplasia is the most prevalent form of skeletal dysplasia, occurring in approximately 1 in 15,000 to 1 in 25,000 live births worldwide [1,2]. This autosomal dominant disorder is primarily caused by mutations in the fibroblast growth factor receptor 3 (*FGFR3*) gene, with the G380R mutation accounting for >95% of cases [3,4]. The condition is characterized by a disproportionate short stature, with affected individuals typically achieving an adult height of 120–145 cm, along with distinctive features, including macrocephaly, frontal bossing, and limb shortening predominantly affecting the proximal segments [5,6].

While extensive research has focused on the medical complications associated with achondroplasia, including spinal stenosis, sleep apnea, and orthopedic concerns [7,8], there remains a significant gap in understanding the functional independence capabilities of individuals with this condition across the lifespan. Previous studies have predominantly examined growth patterns, surgical interventions, and medical management strategies [9,10]. Contemporary clinical practice has been enhanced by evidence-based guidelines that provide comprehensive frameworks for multidisciplinary management across the lifespan [11,12].

Recent research has established WeeFIM as an effective tool for assessing functional capabilities in achondroplasia populations. The foundational work by Ireland et al. [13] provided the methodological groundwork for this field, utilizing WeeFIM-II to evaluate 35 Australian children with achondroplasia and documenting age-related functional patterns. Building upon this foundation, the European LIAISE study [14] represents the largest contemporary multinational investigation. More recent investigations have expanded this evidence base, with the 2023 LIAISE study providing comprehensive multinational data on 108 children and establishing important benchmarks for contemporary functional assessment research [14]. These studies have collectively validated the clinical utility of disease-specific outcome measures in achondroplasia populations [15].

The importance of functional assessment in populations with physical disabilities cannot be overstated because it provides crucial insights for rehabilitation planning, educational accommodations, and family support services [16,17]. The Functional Independence Measure for Children (WeeFIM) has emerged as a gold standard tool for evaluating functional independence in pediatric rehabilitation settings [18,19]. This instrument assesses 18 items across 3 critical domains: self-care activities (i.e., eating, grooming, bathing, dressing, toileting, and continence); mobility functions (i.e., transfers, locomotion, and stairs); and cognitive abilities (i.e., comprehension, expression, social interaction, problem-solving, and memory) [19,20].

Recent advances in understanding achondroplasia have highlighted the importance of early intervention and adaptive strategies to optimize functional outcomes [21,22]. However, the lack of comprehensive functional assessment data has limited the development of evidence-based care protocols and hindered the ability to provide families with realistic expectations regarding their child’s functional potential [23,24]. Furthermore, the recent approval of vosoritide, the first targeted pharmacological treatment for achondroplasia in 2021, has revolutionized therapeutic approaches [25,26]. Clinical trials have demonstrated sustained growth-promoting effects over multiple years of treatment [27,28], with emerging evidence from real-world clinical practice confirming these benefits [29]. The implementation of these novel therapies requires comprehensive monitoring frameworks that extend beyond traditional anthropometric outcomes to include functional independence measures [28].

While previous studies have examined functional capabilities in achondroplasia using various assessment methodologies, comprehensive evaluations using standardized instruments remain limited. This multicenter investigation addresses this gap by providing the first systematic assessment of functional independence in a Taiwanese population of children and adolescents with achondroplasia using the standardized WeeFIM scale.

The primary objective of this study was to establish a comprehensive functional independence database for children and adolescents with achondroplasia, contributing valuable multicenter data from an Asian population to the existing literature. This research pursued four specific aims: first, to evaluate the distribution of functional capabilities across self-care, mobility, and cognitive domains; second, to analyze age-related patterns in functional independence development; third, to identify specific areas of functional challenge that may require targeted interventions; and fourth, to establish baseline functional data to inform clinical care planning and family counseling.

Additionally, this investigation sought to explore relationships between demographic characteristics and functional outcomes, with particular attention to the impact of age at diagnosis and early intervention history. These insights are intended to provide evidence-based guidance for optimizing care delivery strategies within this population.

## 2. Materials and Methods

### 2.1. Study Design and Setting

This multicenter cross-sectional study was conducted between December 2021 and October 2024 across multiple pediatric medical centers in Taiwan (Mackay Memorial Hospital, Chang Gung Memorial Hospital, Kaohsiung Medical University Hospital, National Cheng Kung University Hospital, Taichung Veterans General Hospital, Kaohsiung Veterans General Hospital, Taipei Cathay General Hospital, China Medical University Hospital, National Taiwan University Children’s Hospital, and Taipei Veterans General Hospital).

### 2.2. Participants and Recruitment

The participants were recruited through two primary methods: (1) direct recruitment from pediatric genetics and rehabilitation clinics at participating medical centers and (2) online recruitment through patient advocacy organizations and social media platforms. The inclusion criteria were as follows: individuals who had confirmed diagnosis of achondroplasia based on clinical and/or genetic testing; were aged ≥6 years at the time of assessment; had the ability to participate in functional assessment either independently or with caregiver assistance; and provided informed consent/assent, as appropriate [30,31]. The exclusion criteria included the following: presence of significant cognitive impairment that would preclude valid assessment, concurrent acute medical conditions requiring hospitalization, and incomplete data on core demographic or functional assessment variables [32].

Sample size was calculated based on the previous pediatric functional assessment studies, with an estimated effect size of 0.5, alpha level of 0.05, and power of 0.80, yielding a minimum required sample of 32 participants [33,34]. To account for potential incomplete assessments and ensure adequate representation across age groups, we aimed to recruit 40–50 participants. The final sample size (n = 46) met both the statistical requirements and the target range.

The analysis focused exclusively on participants aged 6–18 years, consistent with the validated age range of the WeeFIM instrument and ensuring appropriate application of this pediatric assessment tool.

### 2.3. Data Collection Procedures

Data were collected through two complementary approaches to maximize participant accessibility and ensure comprehensive coverage. Clinical assessments were conducted by trained healthcare professionals including pediatric physiatrists, occupational therapists, and physical therapists who had received standardized training in WeeFIM administration [35,36]. Online assessments were completed by parents or caregivers using a secure web-based platform that replicated the standard WeeFIM format with detailed instructions and examples [19].

All assessors underwent a standardized training protocol that included the following: (1) review of the WeeFIM administration guidelines, (2) practice assessments using case vignettes, (3) inter-rater reliability testing with experienced WeeFIM administrators, and (4) ongoing quality assurance monitoring throughout the study period [37,38]. The inter-rater reliability was assessed using intra-class correlation coefficients (ICC), with the acceptable reliability defined as an ICC of ≥0.80 for all WeeFIM domains [39].

All WeeFIM assessments were conducted by trained and credentialed examiners per UDSMR requirements. We followed best practices including (1) gathering information from multiple caregivers when available, (2) focusing on typical performance over the preceding 1–2 weeks, (3) requesting specific examples of functional performance, and (4) considering performance across home, school, and community settings. We minimized potential bias from caregiver report through structured interview protocols and verification with multiple informants when possible. While we acknowledge that caregiver report introduces potential bias, this was mitigated through standardized interview protocols.

### 2.4. Outcome Measures

The primary outcome measure was functional independence as assessed using the WeeFIM scale, a validated 18-item instrument designed for individuals aged 6 months to 18 years [40,41]. The WeeFIM scale was applied to all participants within its validated age range of 6–18 years, ensuring appropriate use of this pediatric assessment instrument.

The WeeFIM evaluates three functional domains: self-care (8 items: eating, grooming, bathing, dressing upper body, dressing lower body, toileting, bladder management, and bowel management); mobility (5 items: bed/chair/wheelchair transfers, toilet transfers, tub/shower transfers, locomotion, and stairs); and cognition (5 items: comprehension, expression, social interaction, problem-solving, and memory) [16,41].

Each item is scored on a 7-point ordinal scale: 1 = total assistance (complete dependence), 2 = maximal assistance (75% or more assistance required), 3 = moderate assistance (50–74% assistance), 4 = minimal assistance (25–49% assistance), 5 = supervision or setup (no physical assistance but requires supervision), 6 = modified independence (requires assistive devices but no human assistance), and 7 = complete independence [42,43]. Domain scores range from 8 to 56 for self-care, 5 to 35 for mobility, and 5 to 35 for cognition, with total scores ranging from 18 to 126 [44]. Recent validation studies have confirmed the reliability and validity of WeeFIM across diverse pediatric populations, including children with cerebral palsy and other developmental conditions [45,46].

### 2.5. Demographic and Clinical Variables

Comprehensive demographic and clinical data were collected using a standardized questionnaire developed specifically for this study. The variables included the following: age at assessment; gender; birth date; primary treating hospital and physician; age at initial diagnosis (categorized as infancy [<1 year], early childhood [1–3 years], school age [4–11 years], or adolescence [12–18 years]); genetic testing status and results; current or previous early intervention services (physical, occupational, and speech therapies); educational setting and accommodations; and family socioeconomic characteristics [32,47]. We collected data on pain presence and severity as potential confounding factors, recognizing that up to 90% of adolescents with achondroplasia experience pain that impacts daily functioning.

### 2.6. Statistical Analysis

Participants were stratified into three distinct age groups for comprehensive analysis: early childhood (6–8 years), school age (9–12 years), and adolescence (13–18 years).

Statistical analyses were performed using SPSS version 28.0 (IBM Corp., Armonk, NY) and R version 4.3.0 (R Foundation for Statistical Computing, Vienna, Austria) [48,49]. Descriptive statistics were calculated for all variables, with continuous variables presented as means ± standard deviations or medians with interquartile ranges, as appropriate, based on the distribution normality assessed using the Shapiro–Wilk test [50].

The WeeFIM scores were analyzed as continuous variables and categorized into functional independence levels based on the established cutoff scores, with scores of 6–7 indicating functional independence, scores of 4–5 indicating modified dependence, and scores of 1–3 indicating complete dependence [16,51]. Comparisons with normative data used established WeeFIM norms for typically developing children, consistent with published achondroplasia research. We acknowledge that children with achondroplasia follow unique developmental trajectories, with population-specific norms established by Ireland et al. showing consistent delays of 3–4 standard deviations below typical means [13]. Between-group comparisons were performed using independent *t*-tests for normally distributed continuous variables, Mann–Whitney *U* tests for non-normally distributed variables, and chi-square tests for categorical variables [52].

Age-related patterns in functional independence were examined using Pearson correlation coefficients to assess the relationship between chronological age and WeeFIM domain scores. Participants were stratified into three distinct age groups for comprehensive analysis: early childhood (6–8 years), school age (9–12 years), and adolescence (13–18 years). Multiple linear regression analysis was conducted to identify significant predictors of functional outcomes while controlling for potential confounding variables, including age, gender, age at diagnosis, and early intervention history [53,54]. Statistical significance was established at *p* < 0.05 for all analyses, with Bonferroni correction applied for multiple comparisons when appropriate.

## 3. Results

### 3.1. Participant Characteristics

A total of 46 children and adolescents with confirmed achondroplasia participated in this multicenter cross-sectional study. The demographic characteristics of the study population are presented in Table 1. The cohort included 26 males (56.5%) and 20 females (43.5%), with ages ranging from 6 to 18 years (mean: 13.8 ± 3.6 years). The majority of participants (n = 36, 78.3%) received their initial diagnosis during infancy (before 1 year of age), while 8 participants (17.4%) were diagnosed during early childhood (1–3 years), 1 participant (2.2%) during school age (4–11 years), and 1 participant (2.2%) during adolescence (12–18 years).

Genetic testing was performed in 32 participants (69.6%), with all tested cases confirming mutations in the *FGFR3* gene, predominantly the G380R mutation consistent with typical achondroplasia. Early intervention services were received by 38 participants (82.6%), including physical therapy (n = 35, 76.1%), occupational therapy (n = 32, 69.6%), and speech therapy (n = 26, 56.5%).

### 3.2. WeeFIM Domain Scores

**Figure 1:**
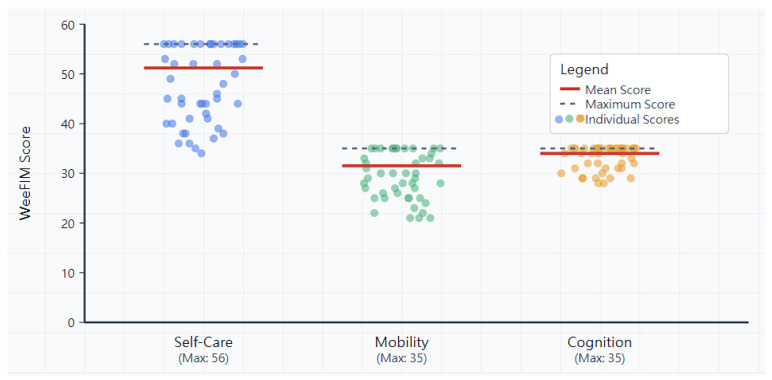
Distribution of WeeFIM scores across three functional domains in 46 children and adolescents with achondroplasia. Each dot represents an individual participant’s score in that domain (n = 46). Horizontal solid lines indicate mean scores for each domain. Horizontal dashed lines show maximum possible scores (Self-care: 56, Mobility: 35, Cognition: 35). Mean scores were highest in cognition (34.0/35, 97.1%), followed by self-care (51.2/56, 91.4%) and mobility (31.5/35, 90.0%). The visualization demonstrates that most children and adolescents with achondroplasia achieved high functional independence across all domains, with cognitive abilities showing the least variability and highest performance.

The functional independence assessment revealed high capability levels across all three WeeFIM domains (Table 2, Figure 1). The mean domain scores were as follows: self-care: 51.2 ± 6.8 out of 56 possible points (91.4% of maximum), mobility: 31.5 ± 4.5 out of 35 possible points (90.0% of maximum), and cognition: 34.0 ± 2.3 out of 35 possible points (97.1% of maximum).

### 3.3. Self-Care Domain

Within the self-care domain, participants demonstrated excellent independence in most activities. Eating (mean: 6.8 ± 0.5), grooming (mean: 6.1 ± 1.4), upper body dressing (mean: 6.3 ± 1.3), and bladder/bowel control (mean: 6.7 ± 0.7 for both) exhibited high functional scores. The greatest challenges were observed in bathing (mean: 5.7 ± 1.9) and toileting activities (mean: 6.0 ± 1.5), where some participants required minimal assistance or adaptive equipment.

### 3.4. Mobility Domain

The mobility domain revealed more variability in functional performance. Chair and toilet transfers showed high independence (mean: 6.4 ± 1.2 and 6.2 ± 1.3, respectively), and locomotion on level surfaces was generally excellent (mean: 6.8 ± 0.4). However, stair climbing was the greatest challenge in this domain (mean: 5.6 ± 1.7), with 17 (37.0%) participants requiring some level of assistance or adaptive strategies. Bathtub transfers also showed reduced independence (mean: 5.4 ± 1.9) compared with other mobility tasks.

### 3.5. Cognitive Domain

The cognitive domain demonstrated remarkably high functional scores across all five items. Most participants showed near-complete independence in comprehension (mean: 6.8 ± 0.5) and memory (mean: 6.7 ± 0.8). Expression abilities (mean: 6.5 ± 1.0), social interaction (mean: 6.6 ± 0.9), and problem-solving skills (mean: 6.1 ± 1.2) were also well-preserved. The majority of participants (93.5%) achieved complete independence in cognitive functions. Only 3 participants (6.5%) exhibited cognitive limitations, which were primarily related to problem-solving in complex situations rather than fundamental cognitive deficits.

### 3.6. Age-Related Patterns

The analysis of functional independence across age groups revealed clear developmental patterns. The total WeeFIM scores showed a strong positive correlation with age (r = 0.74, *p* < 0.001), indicating progressive improvement in functional capabilities from early childhood through adolescence.

**Figure 2:**
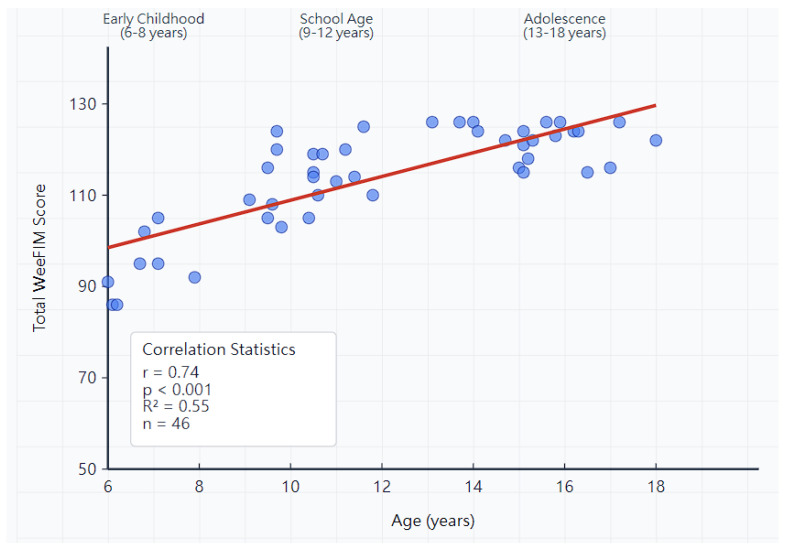
Relationship between participant age and total WeeFIM scores in children and adoles–cents with achondroplasia. Each point represents one child or adolescent participant (n = 46). The fitted regression line demonstrates a strong positive correlation (r = 0.74, *p* < 0.001), indicating progressive improvement in functional independence with increasing age. The steepest functional gains occur during early childhood to school-age transition (ages 6–12 years), with scores continuing to improve through adolescence. This pattern suggests that children and adolescents with achondroplasia continue developing adaptive skills throughout childhood and adolescence, supporting optimistic expectations for functional independence.

The most significant improvements occurred between early childhood and school age, with substantial gains in self-care independence (from 43.8 ± 7.2 to 51.2 ± 5.8, *p* < 0.01) and mobility function (from 26.4 ± 4.8 to 31.8 ± 3.2, *p* < 0.01). Cognitive scores remained consistently high across all age groups, demonstrating that intellectual abilities are not significantly affected by achondroplasia (Figure 2).

### 3.7. Functional Independence Categories

Based on the established WeeFIM criteria for functional independence, participants were categorized according to their ability levels (Table 3). The majority of participants achieved functional independence (scores 6–7) across most domains, with 80.4% demonstrating independence in self-care, 71.7% in mobility, and 93.5% in cognitive functions.

A smaller proportion required supervision only (score 5) without physical assistance: 13.0% for self-care activities, 19.6% for mobility tasks, and 6.5% for cognitive functions. The remaining participants required some level of physical assistance (scores 1–4), representing 6.5% for self-care, 8.7% for mobility, and no participants for cognitive functions.

These findings indicate that while most participants achieved substantial functional independence, specific challenges in mobility and self-care domains required varying levels of support, ranging from environmental modifications to direct assistance for complex tasks.

### 3.8. Factors Associated with Functional Outcomes

The multiple regression analysis identified several factors significantly associated with WeeFIM scores (Table 4). Age at assessment was the strongest predictor of functional independence (β = 0.71, *p* < 0.001), followed by early intervention participation (β = 0.38, *p* = 0.022). Gender did not significantly influence functional outcomes (*p* = 0.412), nor did age at diagnosis (*p* = 0.429), suggesting that functional potential is not substantially affected by the timing of diagnosis within the typical range observed in clinical practice.

Participants who received early intervention services demonstrated higher mean WeeFIM scores compared to those who did not receive such services (115.2 ± 14.8 vs. 104.5 ± 16.2, *p* = 0.039). The beneficial effect was most pronounced in the mobility domain, where early intervention recipients scored significantly higher (32.1 ± 4.1 vs. 27.8 ± 5.4, *p* = 0.018).

The enhanced analysis incorporating therapy timing, duration, and intensity variables reveals that earlier therapy initiation (β = −0.32, *p* = 0.039) and longer duration of physical therapy (β = 0.31, *p* = 0.032) were significantly associated with better functional outcomes, providing clinically meaningful insights for intervention planning. These findings demonstrate that both the timing of intervention initiation and the sustained duration of physical therapy services contribute independently to improved functional independence in children and adolescents with achondroplasia.

Among the 38 participants who received early intervention services, those who began therapy before age 2 years showed superior functional outcomes compared to those who started later (118.3 ± 12.5 vs. 112.1 ± 16.8, *p* = 0.042). The intensity of therapy delivery, measured as sessions per week, showed a positive trend toward improved outcomes but did not reach statistical significance (β = 0.21, *p* = 0.113).

The comprehensive regression model explained 76% of the variance in total WeeFIM scores (R^2^ = 0.76, F = 28.7, *p* < 0.001), indicating robust predictive capability for functional outcomes in this population. These findings underscore the importance of early, sustained intervention programs while highlighting that individual developmental patterns and adaptive capacity remain the primary drivers of functional independence in children and adolescents with achondroplasia.

### 3.9. Specific Challenging Activities

**Figure 3:**
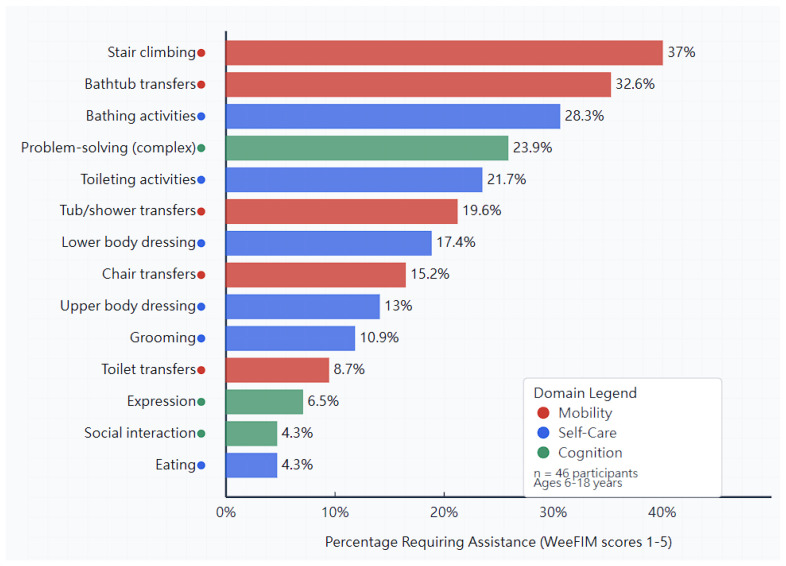
Percentage of children and adolescents requiring assistance for specific WeeFIM. Hori–zontal bar chart showing the percentage of children and adolescents requiring assistance (WeeFIM scores 1–5) for each functional item, ranked from most to least challenging. Mobility-related tasks dominate the most challenging activities, with stair climbing (37.0%) and bathtub transfers (32.6%) presenting the greatest difficulties. These challenges primarily reflect environmental accessibility issues rather than intrinsic functional limitations. Self-care activities generally show high independence rates, with eating requiring assistance in only 4.3% of participants, demonstrating preserved fine motor and cognitive abilities in children and adolescents with achondroplasia.

A detailed item analysis, shown in Figure 3, revealed the specific activities that consistently presented challenges across the cohort. The five most challenging tasks, in order of difficulty, were stair climbing (37.0% requiring some level of assistance), bathtub transfers (32.6% requiring assistance), bathing activities (28.3% requiring assistance), problem-solving in complex situations (23.9% showing some limitation), and toileting activities (21.7% requiring assistance or adaptive equipment).

Individual item scores within these challenging activities ranged from 3 to 6 on the WeeFIM scale, indicating that while some participants achieved independence with modifications, others required varying degrees of assistance. Notably, no participant scored below 3 (moderate assistance) on any individual item, suggesting that fundamental capabilities remained intact across the cohort.

These challenges were primarily attributed to environmental barriers and physical accessibility issues rather than intrinsic functional limitations. Most difficulties could be effectively addressed through environmental modifications, adaptive equipment, or alternative strategies that maintained functional independence while ensuring safety and efficiency.

## 4. Discussion

This multicenter cross-sectional study contributes to the growing body of evidence on functional independence in achondroplasia, building upon the methodological foundation established by Ireland et al. [13] and complementing recent findings from the European LIAISE study [14]. Our results demonstrate functional independence levels consistent with these previous investigations while providing novel insights from an Asian population perspective focusing exclusively on children and adolescents within the validated age range of the WeeFIM assessment tool.

### 4.1. Cognitive Function Preservation

The cognitive domain showed the highest functional scores (34.0 ± 2.3 of 35 points), with 93.5% of participants achieving complete independence. While the cognitive domain showed the highest functional scores, we must interpret these findings cautiously. WeeFIM, as a functional independence measure, primarily assesses the application of cognitive skills in daily living rather than comprehensive neuropsychological evaluation. Published neuropsychological studies have indeed identified subtle challenges in specific cognitive domains among individuals with achondroplasia, including executive function and processing speed difficulties [55,56]. Our findings suggest that functional cognitive abilities remain largely intact, but this should not be interpreted as excluding the possibility of more nuanced cognitive differences.

While our WeeFIM cognitive scores were high, specialized neuropsychological studies have reported specific challenges in executive function, attention, and processing speed among individuals with achondroplasia [55,56]. These findings emphasize the importance of using comprehensive assessment approaches rather than relying solely on functional measurement tools.

This finding generally aligns with the previous literature establishing that achondroplasia does not fundamentally affect intellectual development [5,6], though our results must be interpreted within the context of WeeFIM’s functional focus rather than comprehensive cognitive assessment. Contemporary research has reinforced the importance of using validated functional assessment tools while acknowledging their limitations in detecting subtle cognitive differences that may require specialized neuropsychological evaluation [46].

### 4.2. Mobility Challenges and Adaptive Strategies

The mobility domain revealed the most variability in functional performance, with specific challenges in stair climbing (37.0% requiring assistance) and bathtub transfers (32.6% requiring assistance). These findings are consistent with the known anatomical features of achondroplasia, including shorter limbs and altered joint mechanics that can affect certain mobility tasks [23,24]. However, the overall mobility domain score (90.0% of maximum) demonstrates that most mobility challenges can be effectively managed through adaptive strategies and environmental modifications.

The age-related improvement in mobility scores from early childhood (26.4 ± 4.8) to adolescence (33.2 ± 2.1) suggests that children and adolescents with achondroplasia develop increasingly effective compensatory mechanisms over time. This developmental pattern has important implications for intervention planning because early mobility training and adaptive equipment provision may facilitate optimal functional outcomes [57,58].

Notably, locomotion on level surfaces showed excellent independence (mean: 6.8 ± 0.4), indicating that basic mobility is well-preserved. The specific challenges associated with stair climbing and bathtub transfers likely reflect environmental barriers rather than intrinsic functional limitations, suggesting that architectural modifications and assistive devices can substantially improve independence in these areas.

These findings align with the patterns identified in Ireland’s longitudinal study [13], which documented similar challenges in mobility tasks among Australian children with achondroplasia. The consistency of these functional patterns across different populations and healthcare systems supports the universal nature of these challenges and validates the cross-cultural applicability of our findings. Recent systematic reviews have confirmed that pain prevalence in achondroplasia increases with age, affecting up to 75% of adults and significantly impacting functional independence and daily activities [59,60]. Understanding these pain-related functional limitations is crucial for interpreting WeeFIM scores in the context of real-world experiences [61].

Pain represents a significant confounding factor in our functional assessments. Published evidence shows pain prevalence increases with age in achondroplasia, affecting physical, emotional, social, and school functioning. While we cannot definitively separate pain-related functional limitations from those directly attributable to skeletal dysplasia, this reflects the real-world experience of individuals with achondroplasia where pain and physical limitations are interrelated.

### 4.3. Self-Care Independence and Development

The self-care domain demonstrated high overall independence (91.4% of maximum), with age-related improvements from early childhood through adolescence. While our study demonstrates relatively high functional independence levels, children with achondroplasia do show delays in achieving developmental milestones compared to age-matched typically developing peers, particularly in self-care skills during early childhood. This developmental trajectory parallels typical functional development, suggesting that children with achondroplasia acquire self-care skills following normal developmental sequences, albeit with delays related to physical adaptations. Ireland et al. demonstrated that children with achondroplasia required significantly greater caregiver assistance for self-care activities compared to typically developing peers, with delays persisting across ages 3–7 years [13]. Pfeiffer et al. provided specific evidence that at age 7, many children still require parental assistance with basic self-care including dressing, toileting, bathing, and grooming [62].

Within the self-care domain, eating and grooming showed the highest independence levels, consistent with the preserved fine motor skills in achondroplasia. The relatively greater challenges in bathing (mean: 5.7 ± 1.9) and toileting (mean: 6.0 ± 1.5) likely reflect height-related accessibility issues rather than fundamental skill deficits. These findings highlight the importance of ensuring bathroom modifications and introducing adaptive equipment to promote independence.

The strong correlation between age and self-care scores (r = 0.74, *p* < 0.001) indicates continued skill acquisition throughout childhood and adolescence. This pattern suggests that families and clinicians should maintain optimistic expectations for functional independence while providing appropriate support during skill development phases.

### 4.4. Impact of Early Intervention Services

Our analysis revealed significant associations between early intervention participation and functional outcomes, particularly in the mobility domain. Participants who received early intervention services demonstrated higher mean WeeFIM scores (115.2 ± 14.8 vs. 104.5 ± 16.2, *p* = 0.039), with the most pronounced benefits in mobility function. This finding supports the current clinical practice recommendations for comprehensive early intervention programs for children with achondroplasia [30,31].

The beneficial effects of physical, occupational, and speech therapies align with the evidence from other pediatric populations with physical disabilities, wherein early intervention has been shown to optimize developmental trajectories [16,17]. Our results suggest that systematic early intervention programs specifically designed for children with achondroplasia may enhance long-term functional outcomes, particularly in mobility-related activities.

Notably, 82.6% of participants in our study received early intervention services, reflecting the current clinical awareness of the potential benefits of these programs. The high proportion of participants receiving multiple intervention modalities (76.1% physical therapy, 69.6% occupational therapy, and 56.5% speech therapy) indicates a comprehensive approach to addressing the multifaceted needs of children with achondroplasia.

### 4.5. Environmental Factors and Accessibility

The specific challenges identified in our study—stair climbing, bathtub transfers, and certain self-care activities—predominantly reflect environmental barriers rather than intrinsic functional limitations. This distinction is crucial for intervention planning because environmental modifications and adaptive equipment can often completely eliminate these barriers to independence.

The high overall functional independence scores achieved by participants in our study suggest that with appropriate accommodations, children and adolescents with achondroplasia can achieve near-normal functional capabilities. This finding has important implications for school accommodation planning, supporting the view that environmental modifications are often more effective than direct personal assistance in promoting independence [8,23].

Our results also highlight the importance of considering the built environment in functional assessment. Standard environments designed for individuals with average height may falsely reduce the functional independence scores of individuals with achondroplasia, emphasizing the need for universal design principles to improve accessibility in educational and public spaces.

### 4.6. Developmental Functional Patterns

The strong positive correlation between age and total WeeFIM scores (r = 0.74, *p* < 0.001) demonstrates continued functional improvement throughout childhood and adolescence. This developmental pattern indicates that children and adolescents with achondroplasia continue refining adaptive strategies as they mature, with the most significant gains occurring during the transition from early childhood to school age.

The most significant functional gains occurred between early childhood and school age, with continued improvements through adolescence. This pattern has important implications for family counseling and educational planning, as functional capabilities continue to improve throughout the school years. The persistence of functional gains through adolescence suggests that long-term functional prognosis within the pediatric years is generally favorable.

### 4.7. Clinical Implications and Future Directions

The findings of this study have several important clinical implications. First, the high functional independence scores encourage optimistic counseling for families of children with achondroplasia, while acknowledging the specific areas where support and adaptation may be needed. Second, the identification of specific challenging activities provides targets for focused intervention and accommodation planning.

The strong association between early intervention and functional outcomes supports the current recommendations for comprehensive multidisciplinary care from early childhood [30,31]. Recent comprehensive reviews of achondroplasia burden have identified functional limitations as key targets for intervention, with studies documenting significant impacts on daily activities and quality of life across diverse populations [63,64]. Real-world evidence from emerging therapeutic interventions, including vosoritide treatment, demonstrates measurable improvements in growth parameters and suggests potential benefits for long-term functional outcomes [29,65]. However, our results also suggest that intervention programs should be specifically tailored to address the unique functional challenges associated with achondroplasia, particularly mobility-related activities and environmental adaptations.

Future research should investigate the long-term effectiveness of specific intervention strategies and the optimal timing and intensity of therapeutic services. Longitudinal studies following children with achondroplasia into adulthood would provide valuable insights into the persistence of functional gains and the transition to adult independence. Studies examining the relationship between functional independence and quality of life outcomes are needed to provide comprehensive insights for clinical care planning.

### 4.8. Study Limitations

Several important limitations must be considered when interpreting the results of this study. These limitations can be categorized into methodological, sampling, and instrument-related concerns.

a. Methodological Limitations:

The cross-sectional design restricts our ability to establish causal inferences regarding developmental trajectories, though the strong age correlations provide compelling evidence for functional improvement over time. Additionally, the absence of a control group with typically developing peers limits our ability to contextualize functional independence levels within normative expectations.

b. Sampling and Generalizability Concerns:

Multiple factors may limit the generalizability of our findings. The participant sample was recruited primarily from specialized medical centers in Taiwan, which may not represent the broader population of individuals with achondroplasia who do not access specialized care or reside in different healthcare systems with varying cultural backgrounds and socioeconomic conditions. The relatively small sample size of 46 participants, while meeting statistical power requirements, reflects the inherent challenges of studying rare conditions and may further restrict generalizability. Potential selection bias exists due to the exclusion of individuals with acute medical conditions, which may have inadvertently excluded patients with concurrent but unrelated conditions that would not affect functional assessment.

c. Assessment Tool Appropriateness:

This study appropriately applied the WeeFIM instrument within its validated age range of 6–18 years, ensuring developmentally appropriate functional assessment. The exclusive focus on children and adolescents allows for meaningful interpretation of results within the established normative framework of the instrument.

The WeeFIM cognitive domain assesses functional cognitive skills rather than comprehensive neuropsychological abilities. This tool may not detect subtle cognitive differences that would require specialized neuropsychological testing to identify.

We acknowledge that pain may have influenced WeeFIM scores, particularly in self-care and mobility domains. Future studies should incorporate validated pain assessment tools alongside functional measures to better understand these relationships.

d. Data Collection Variability:

The assessment methods incorporated both clinical evaluations and parent-reported outcomes, which may have introduced variability in scoring accuracy. However, standardized training protocols and acceptable inter-rater reliability coefficients exceeding 0.80 help mitigate these concerns.

These limitations collectively suggest that while our findings provide valuable insights into functional independence among children and adolescents with achondroplasia using an appropriately applied assessment instrument, future research incorporating larger sample sizes and longitudinal designs would strengthen the evidence base for clinical decision-making.

## 5. Conclusions

This study provides comprehensive functional independence data for children and adolescents with achondroplasia using a standardized measurement tool, contributing valuable baseline data from a multicenter Taiwanese cohort of 46 children and adolescents to the existing literature on functional capabilities in this population. The high levels of functional independence achieved across all domains, combined with clear developmental progression patterns through adolescence, support an optimistic long-term functional prognosis while identifying specific areas where targeted interventions and environmental modifications can enhance independence.

The preserved cognitive function, mobility skill developments, and age-appropriate self-care capabilities demonstrate that achondroplasia, while presenting specific challenges, does not fundamentally impair functional independence in daily living activities during childhood and adolescence. These findings provide valuable baseline data for clinical care planning, family counseling, and the development of evidence-based intervention strategies tailored to the unique needs of individuals with achondroplasia.

Our results emphasize the importance of comprehensive early intervention services, environmental accessibility modifications, and individualized support strategies for optimizing functional outcomes. The demonstration of continued functional improvement into young adulthood suggests that intervention benefits may persist through the pediatric years. As new therapeutic interventions for achondroplasia continue to emerge, these functional independence measures provide important baseline data for evaluating treatment efficacy beyond traditional anthropometric outcomes.

The identification of specific challenging activities, particularly stair climbing and bathtub transfers, provides clear targets for environmental modifications and recommendations for adaptive equipment. The strong association between early intervention services and improved functional outcomes supports the continued emphasis on comprehensive multidisciplinary care from early childhood through adulthood.

## Figures and Tables

**Table 1:** Demographic and clinical characteristics of the study participants (*n* = 46).

Characteristic	n (%) or Mean ± SD
**Age at assessment (years)**	13.8 ± 3.6
Range	6–18
**Age groups**	
Early childhood (6–8 years)	8 (17.4)
School age (9–12 years)	18 (39.1)
Adolescence (13–18 years)	20 (43.5)
**Gender**	
Male	26 (56.5)
Female	20 (43.5)
**Age at diagnosis**	
**Infancy (<1 year)**	36 (78.3)
**Early childhood (1–3 years)**	8 (17.4)
**School age (4–11 years)**	1 (2.2)
**Adolescence (12–18 years)**	1 (2.2)
**Genetic testing performed**	32 (69.6)
***FGFR3* mutation confirmed**	32 (100.0)
**Early intervention services**	38 (82.6)
**Physical therapy**	35 (76.1)
**Occupational therapy**	32 (69.6)
**Speech therapy**	26 (56.5)
**Early intervention details**	
Age at therapy initiation (years)	2.9 ± 1.5
Duration of physical therapy (months)	29.2 ± 15.8
Duration of occupational therapy (months)	24.8 ± 13.2
Duration of speech therapy (months)	19.1 ± 12.0
Therapy intensity (sessions/week)	2.4 ± 0.9
**Primary indication for therapy referral**	
Developmental delay	29 (76.3)
Motor skill enhancement	26 (68.4)
Speech delay	16 (42.1)
Adaptive equipment training	14 (36.8)
Multiple indications	20 (52.6)

Note: Data presented as mean ± standard deviation for continuous variables and frequency (percentage) for categorical variables. Early intervention details calculated for participants who received services (n = 38). *FGFR3* = Fibroblast Growth Factor Receptor 3.

**Table 2:** WeeFIM domain and total scores by age group.

Age Group	n	Self-Care	Mobility	Cognition	Total WeeFIM
**Early childhood (6–8 years)**	8	43.8 ± 7.2	26.4 ± 4.8	32.1 ± 2.9	98.3 ± 15.2
**School age (9–12 years)**	18	51.2 ± 5.8	31.8 ± 3.2	33.8 ± 1.8	114.8 ± 12.6
**Adolescence (13–18 years)**	20	54.1 ± 4.2	33.2 ± 2.1	34.9 ± 1.2	121.4 ± 8.3
**Overall**	46	51.2 ± 6.8	31.5 ± 4.5	34.0 ± 2.3	113.7 ± 15.2
**F-statistic (*p*-value)**		14.67 (<0.001)	18.43 (<0.001)	10.28 (<0.001)	21.85 (<0.001)

Note: Data presented as mean ± standard deviation. Maximum possible scores: Self-care = 56, Mobility = 35, Cognition = 35, Total WeeFIM = 126. Statistical analysis performed using one-way ANOVA with post hoc Tukey tests for between-group comparisons. All pairwise comparisons between age groups were statistically significant (*p* < 0.05) except for cognition scores between school age and adolescence groups (*p* = 0.082).

**Table 3:** Functional independence categories in WeeFIM domains.

Independence Level	Self-Care n (%)	Mobility n (%)	Cognition n (%)
**Complete independence (6–7)**	37 (80.4)	33 (71.7)	43 (93.5)
**Supervision (5)**	6 (13.0)	9 (19.6)	3 (6.5)
**Complete dependence (1–4)**	3 (6.5)	4 (8.7)	0 (0.0)

Note: Functional independence categories based on established WeeFIM criteria. Complete independence (scores 6–7) indicates ability to perform activities safely and effectively without assistance. Supervision (score 5) indicates need for cueing, coaxing, or monitoring but no physical assistance. Complete dependence (scores 1–4) indicates requirement for physical assistance ranging from minimal to total help. Data presented as frequency and percentage of total sample (n = 46).

**Table 4:** Multiple regression analysis: factors associated with the total WeeFIM scores.

Variable	β Coefficient	Standard Error	t-Value	*p*-Value	95% CI
**Age at assessment**	0.71	0.11	6.45	<0.001	0.49–0.93
**Early intervention**	0.38	0.16	2.38	0.022	0.06–0.70
**Gender (male)**	0.15	0.18	0.83	0.412	−0.21–0.51
**Age at diagnosis**	−0.12	0.15	−0.80	0.429	−0.42–0.18
**Genetic testing**	0.09	0.17	0.53	0.601	−0.25–0.43
**Age at therapy initiation**	−0.32	0.15	−2.13	0.039	−0.62–0.02
**Duration of physical therapy**	0.31	0.14	2.21	0.032	0.03–0.59
**Duration of occupational therapy**	0.19	0.16	1.19	0.241	−0.13–0.51
**Therapy intensity**	0.21	0.13	1.62	0.113	−0.05–0.47
**Multiple therapy indications**	0.28	0.17	1.65	0.107	−0.06–0.62

R^2^ = 0.76, Adjusted R^2^ = 0.73, F = 28.7, *p* < 0.001. Note: Multiple linear regression analysis with total WeeFIM score as the dependent variable. β coefficients represent standardized regression coefficients. CI = Confidence Interval. Model includes all children and adolescents aged 6–18 years (n = 46). Therapy-related variables calculated for participants who received early intervention services (n = 38). Significant predictors (*p* < 0.05) include age at assessment, early intervention participation, age at therapy initiation, and duration of physical therapy.

## Data Availability

Complete datasets supporting this research are included within the published article.
